# Identification of inflammation-related biomarkers in keloids

**DOI:** 10.3389/fimmu.2024.1351513

**Published:** 2024-02-20

**Authors:** Xiaochuan Wang, Xiaoyang Wang, Zhenzhong Liu, Lei Liu, Jixun Zhang, Duyin Jiang, Guobao Huang

**Affiliations:** ^1^ Plastic Burn Surgery, The Second Hospital of Shandong University, Jinan, Shandong, China; ^2^ Burn Plastic Surgery, Central Hospital Affiliated to Shandong First Medical University, Jinan, Shandong, China

**Keywords:** inflammation-related genes, keloid disease, GEO, alignment diagram, biomarker

## Abstract

**Background:**

The relationship between inflammation-related genes (IRGs) and keloid disease (KD) is currently unclear. The aim of this study was to identify a new set of inflammation-related biomarkers in KD.

**Methods:**

GSE145725 and GSE7890 datasets were used in this study. A list of 3026 IRGs was obtained from the Molecular Signatures Database. Differentially expressed inflammation-related genes (DEGs) were obtained by taking the intersection of DEGs between KD and control samples and the list of IRGs. Candidate genes were selected using least absolute shrinkage and selection operator (LASSO) regression analysis. Candidate genes with consistent expression differences between KD and control in both GSE145725 and GSE7890 datasets were screened as biomarkers. An alignment diagram was constructed and validated, and in silico immune infiltration analysis and drug prediction were performed. Finally, RT-qPCR was performed on KD samples to analyze the expression of the identified biomarkers.

**Results:**

A total of 889 DEGs were identified from the GSE145725 dataset, 169 of which were IRGs. Three candidate genes (*TRIM32*, *LPAR1* and *FOXF1*) were identified by the LASSO regression analysis, and expression validation analysis suggested that *FOXF1* and *LPAR1* were down-regulated in KD samples and *TRIM32* was up-regulated. All three candidate genes had consistent changes in expression in both the GSE145725 and GSE7890 datasets. An alignment diagram was constructed to predict KD. Effector memory CD4 T cells, T follicular helper cell, Myeloid derived suppressor cell, activated dendritic cell, Immature dendritic cell and Monocyte were differentially expressed between the KD and control group. Sixty-seven compounds that may act on *FOXF1*, 108 compounds that may act on *LPAR1* and 56 compounds that may act on *TRIM32* were predicted. Finally, RT-qPCR showed that the expression of *LPAR1* was significantly lower in KD samples compared to normal samples whereas *TRIM32* was significantly higher, while there was no difference in the expression of *FOXF1*.

**Conclusion:**

This study provides a new perspective to study the relationship between IRGs and KD.

## 1 Introduction

Keloid disease (KD) is a benign skin fibroplasia caused by abnormal wound healing after skin injury (1) leading to hyperplasic invasive growth, and has a high recurrence rate (2). The occurrence of KD involves trauma, chronic inflammation, and fibrosis tumor inheritance (1, 3, 4). Keloids can grow on all parts of the body (5), and are accompanied by unbearable itching and pain which seriously affects quality of life. Keloids, especially on the face, can also have a serious impact on mental health (6, 7). Although there are many studies on KD the pathogenesis is still not completely clear (8); improved understanding of the pathogenesis will likely lead to new treatments. Several studies have shown that inflammation is involved in regulating KD collagen synthesis, and the intensity of inflammation is positively correlated with the final scar size (9, 10). Therefore, study of the inflammation-related molecular pathogenesis of KD may lead to new KD prevention and treatment strategies.

It is well known that scars are the result of both inflammation and fibrosis after injury repair (1, 8, 11). In the early stage of repair, inflammatory cells play a pro-inflammatory role through cytokines. It usually enters the repair and healing stage after 72 hours and finally completes the remodeling of collagen (12). Pro-inflammatory factors such as IL-1α, IL-1β, IL-6 and TNF-α are up-regulated in KD tissue (11). It has been speculated that chronic inflammation persists in KD causing excessive deposition of extracellular matrix which is an important cause of keloid formation (13, 14). This indicates that KD is an inflammatory disease of the skin (12). In addition, Shi et al. demonstrated that IL-10 can negatively regulate collagen synthesis, thereby reducing scar formation (13, 15). Nishiguchi et al. reported that the chemokine CXCL12 can promote scar formation in mice (12, 16). A large number of studies have shown that KD is correlated with chronic inflammation (11, 12, 17). However, few studies have explored of inflammation-related genes IRGs in KD and the specific mechanism of action in KD pathogenesis. Therefore, we identified and analyzed differentially-expressed IRGs in KD in order to discover new genes that might be important in KD pathogenesis, both as biomarkers for early diagnosis and as novel drug targets.

## 2 Materials and methods

### 2.1 Data source

Two KD datasets (GSE145725 and GSE7890) were obtained from the Gene Expression Omnibus (GEO) database (https://www.ncbi.nlm.nih.gov/gds). The GSE145725 dataset contains 9 fibroblast samples from KD and 10 normal fibroblast control samples. The GSE7890 dataset contains 5 fibroblast samples from KD and 5 normal fibroblast control samples. IRGs were obtained from the Molecular Signatures Database (MSigDB, https://www.gsea-msig) by using the search term “INFLAMMATORY”. A total of 57 fibrosis-related genes were shown in **Supplementary Table 1**.

### 2.2 Identification of inflammation-related DEGs

Differential expression analysis was performed between KD and control samples in the GSE145725 dataset using the limma R package (18) to screen differentially expressed genes (DEGs) using cutoffs of |log_2_FC| > 0.5 and adj. P < 0.05. Gene ontology (GO) and Kyoto encyclopedia of genes and genomes (KEGG) enrichment analyses of DEGs were completed using the clusterProfiler package (19). Inflammation-related DEGs were obtained by taking the intersection of DEGs and IRGs. To explore whether interactions existed among the inflammation-related DEGs, a protein-protein interaction (PPI) network was created using STRING (https://string-db.org).

### 2.3 Acquisition of biomarkers

To obtain candidate genes, least absolute shrinkage and selection operator (LASSO) regression analysis SVM, and Boruta algorithms were performed on the inflammation-related DEGs using the glmnet (20), e1071 and Boruta packages. In addition, candidate genes were validated by checking that they were also differentially expressed in the GSE7890 dataset. Validated candidate genes were screened as biomarkers. To explore the potential mechanisms of the biomarkers, Gene Set Enrichment Analysis (GSEA) of biomarkers in GSE145725 was conducted using the h.all.v2023.1.Hs.symbols.gmt dataset in the clusterProfiler package (19). Differential analysis of fibrosis-related genes in the GSE145725 dataset and correlation analysis of differential fibrosis-related genes with biomarkers to further explore the function of biomarkers.

### 2.4 Construction and validation of alignment diagram

To predict the probability of KD from the expression of the identified biomarkers, an alignment diagram was constructed using the rms package (21) in R. In order to assess the predictive ability of the alignment diagram, a calibration curve was plotted using the calibrate function in the rms package, where the closer the slope is to 1, the more accurate the prediction. In order to evaluate the clinical effectiveness of the alignment diagram, decision curve analysis (DCA) was performed using the “rmda” package. Based on the DCA curve, the clinical impact curve (CIC) was plotted using the model to predict the risk stratification of 1000 people.

### 2.5 Immuno-infiltration analysis and drug prediction

The immune abundance of 28 immune cells in KD and control samples from GSE145725 was calculated using the ssGSEA algorithm (22) to obtain differentially expressed (DE) immune cells, and the correlation between the ssGSEA scores of DE immune cells and biomarkers was calculated and presented using a heatmap. Compounds that may act on biomarkers were predicted using the Comparative Toxicogenomics Database (CTD) database (http://ctdbT2Dme.org/) and key gene-compound networks were constructed.

### 2.6 Protein expression analysis of biomarkers and construction of miRNA-mRNA-TFs regulatory network

The expression of the identified biomarkers was analyzed in different human skin tissues using the Bgee database (https://bgee.org/). To further explore their expression in different cell types of the skin, the Human Protein Atlas (http://www.proteinatlas.org/) was used. The miRNAs that may target the identified biomarkers were predicted using the MicroRNA Target Prediction Database (miRDB, https://mirdb.org/) and The Encyclopedia of RNA Interactomes (ENCORI, http://starbase.sysu.edu.cn/index.php), and the intersection of the predictions from the two databases was taken as the candidate miRNA. Transcription factors (TF) that regulate the expression of the identified biomarkers were predicted using the NetworkAnalyst online tool (https://www.networkanalyst.ca/ and hTFtarget database (http://bioinfo.life.hust.edu.cn). Finally, miRNA-mRNA-TF regulatory networks were constructed using Cytoscape.

### 2.7 Statistical analysis

The limma package was used to identify DEGs. Venn diagrams were constructed using the venn package. ClusterProfiler was used for enrichment analysis. STRING was used to build PPI networks. LASSO was used to screen candidate genes. ssGSEA was used to calculate the infiltration abundance of immune cells. Statistical analysis was done using R software (version 4.1.1 https://www.r-project.org/). Differences between groups were analyzed using the Wilcox test. P < 0.05 was considered a statistically significant difference.

### 2.8 RT-qPCR Analysis

The expression of the three biomarkers was measured using RT-qPCR. We collected KD and control samples from The Second Hospital of Shandong University department of plastic surgery with 5 samples in each group. This study was performed in line with the principles of the Declaration of Helsinki. Approval was granted by the Ethics Committee of the Second Hospital of Shandong university(Date: December 6, 2023; No: KYLL-2023LW088). Total RNA was extracted using TRIzol (Ambion, Austin USA) according to the manufacturer’s instructions. The extracted RNA was reverse transcribed into cDNA using the SureScript First strand cDNA synthesis kit before RT-qPCR. RT-qPCR was performed using the 2xUniversal Blue SYBR Green qPCR Master Mix (Servicebio, Wuhan China). The *GAPDH* gene was used as a housekeeping gene and the relative expression of the biomarkers was determined using the 2^-ΔΔCt^ method.

## 3 Results

### 3.1 Identification of inflammation-associated DEGs in the GSE145725 dataset

**Figure 1:**
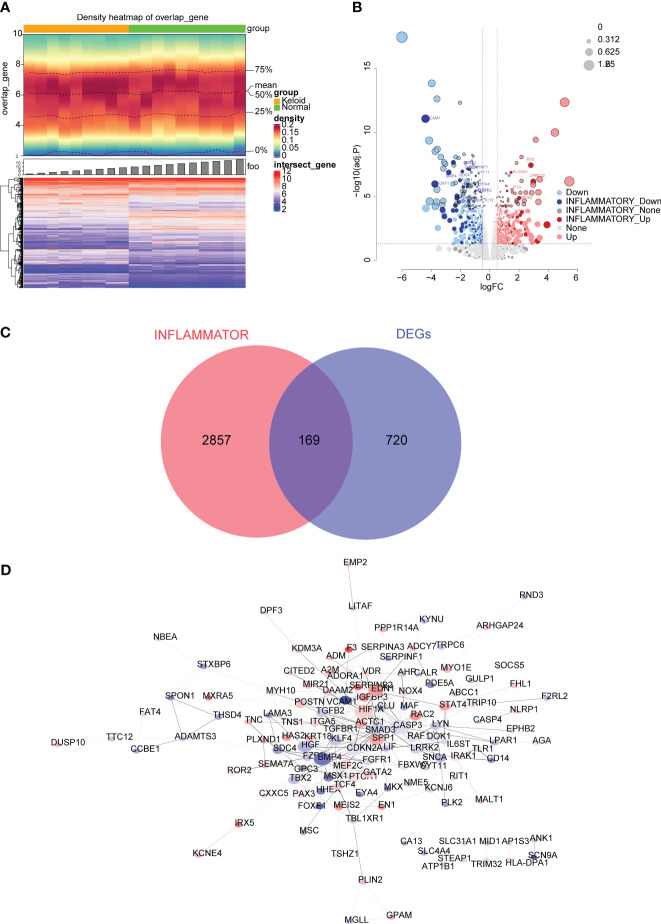
Differential expression analysis in the GSE145725 dataset. **(A)** Heatmap of differentially expressed genes (DEGs) between keloid disease (KD) and normal samples. A heat map of gene density is shown at the top, and a heat map of gene expression is shown at the bottom (red is high expression, blue is low expression). **(B)** Volcano plot of DEGs between KD and normal groups. Each dot represents a gene, the darker colored dots indicate inflammation-related genes, and the black circles indicate genes with an adjusted P value < 0.01. The names of genes associated with inflammation with very significant differences are labeled in the figure. **(C)** Venn diagram of 169 inflammation-related DEGs obtained by overlapping the DEGs and inflammation-related genes (IRGs). **(D)** Protein-protein interaction (PPI) network of 169 inflammation-associated DEGs.

A total of 889 DEGs were identified from the GSE145725 dataset, of which 433 were up-regulated in KD and 456 were down-regulated (**Figures 1A, B**). GO analysis revealed that DEGs were associated with skeletal system morphogenesis, regulation of animal organ morphogenesis, and cartilage development (**Supplementary Figure 1A**) and KEGG analysis revealed enriched in transcriptional misregulation in cancer, cGMP-PKG signaling pathway, and Wnt signaling pathway (**Supplementary Figure 1B**). A total of 169 inflammation-related DEGs were obtained from the overlap between the 889 DEGs and 3026 IRGs (**Figure 1C**). To explore whether there are any known interactions between the proteins coded for by the 169 inflammation-associated DEGs, a PPI network was created (**Figure 1D**) which had a confidence level of 0.4 (Confidence = 0.4) with strong interactions between A2M and SERPINF1, ABCC1 and CASP3, and ADAMTS3 and TTC12.

### 3.2 Screening and verification of biomarkers for KD

**Figure 2:**
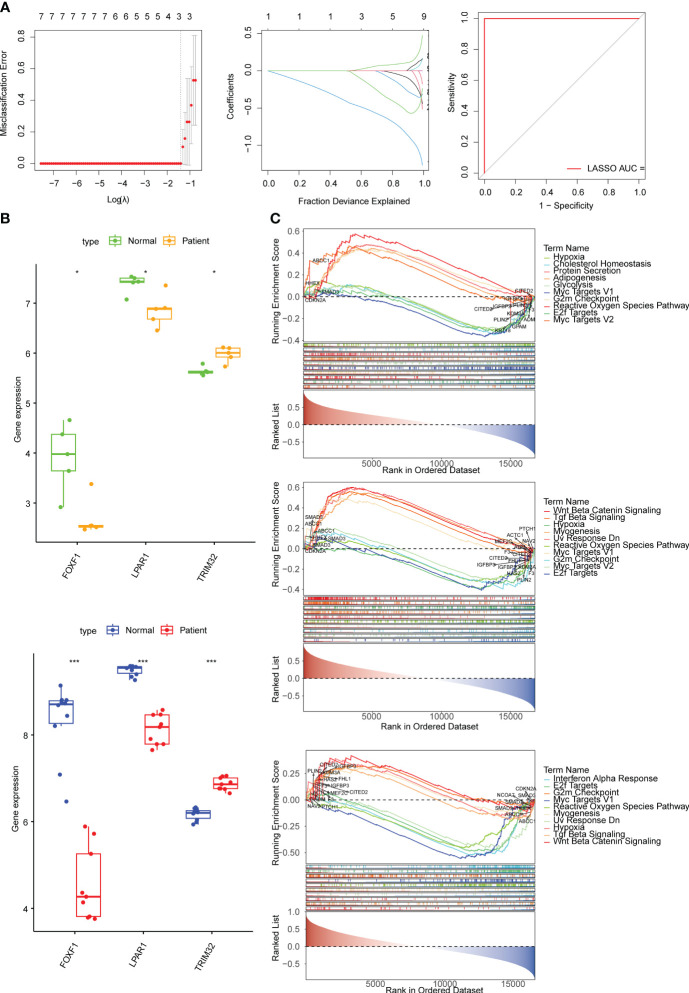
Identification of biomarkers and exploration of potential function. **(A)** Error plots for 10-fold cross-validation, plot of gene coefficients, and receiver operating characteristic (ROC) curve of the least absolute shrinkage and selection operator (LASSO) model. The different colored lines represent different genes. AUC, area under the curve. **(B)** The expression of biomarkers in the KD and normal samples in the GSE7890 and GSE145725 datasets. **(C)** The top 10 pathways significantly enriched in *FOXF1*, *LPAR1*, and *TRIM32* according to gene set enrichment analysis (GSEA) enrichment analysis. * means p<0.05, *** means p<0.001.

FOXF1, LPAR1, SERPINF1, TRIM32 were found as candidate genes by machine learning (SVM and Boruta) (**Supplementary Figure 2A**). The results of the LASSO regression analysis suggested that when λ = 0.004102608 three candidate genes (*TRIM32*, *LPAR1*, and *FOXF1*) with regression coefficients that were not penalized to 0 were obtained after tenfold cross-validation (**Figure 2A**). *FOXF1* and *LPAR1* were down-regulated in KD samples and *TRIM32* was up-regulated in KD samples and all three candidate genes had the same expression trends in the GSE145725 and GSE7890 datasets (**Figure 2B**). GSEA results showed that *FOXF1* was mainly enriched in E2f targets, G2M checkpoint, and myogenesis. *LPAR1* was mainly enriched in reactive oxygen species pathway, apoptosis, and IFN-α response. *TRIM32* was mainly enriched in IFN-α response, apoptosis, and hypoxia (**Figure 2C**). Correlation analysis showed that nine fibrosis-related genes were significantly different between KD and controls and showed high correlation with biomarkers (**Supplementary Figure 2B**).

### 3.3 Prediction of KD risk from biomarker expression

**Figure 3:**
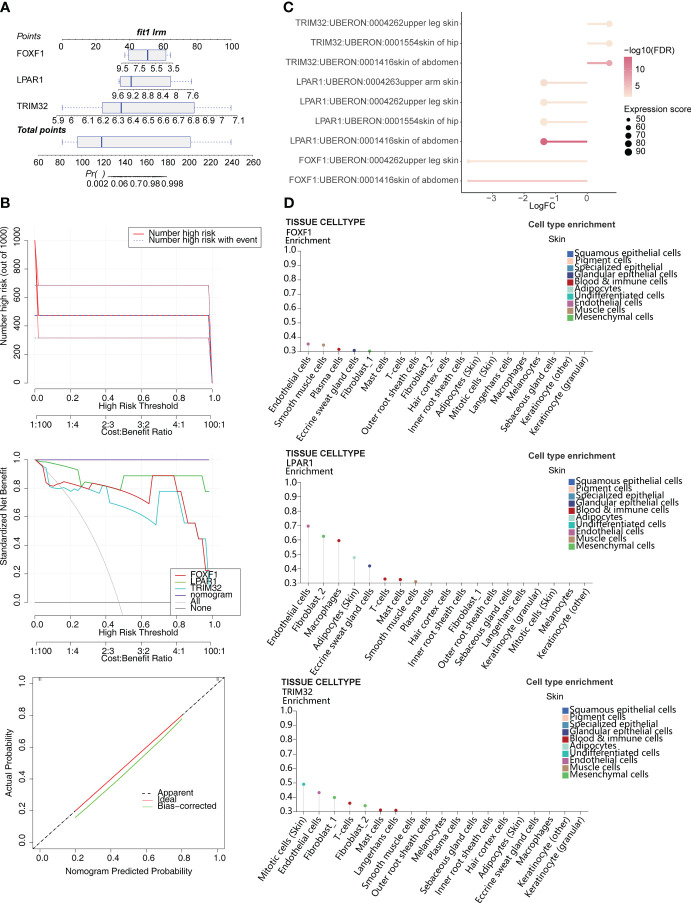
Construction of the alignment diagram to predict the risk of KD. **(A)** Alignment diagram based on expression of *FOXF1*, *LPAR1*, and *TRIM32*. **(B)** Clinical impact curve (CIC), decision curve analysis (DCA), and calibration curve of the alignment diagram. **(C)** Distribution of biomarkers in human tissues. **(D)** Expression of biomarkers in different skin cell types.

Based on the expression of the biomarkers, an alignment diagram was constructed. The score of each sample was calculated by the alignment diagram, with a higher score indicating a higher likelihood of KD (**Figure 3A**). The slope of the calibration curve is close to 1 and the CIC converge with the trend of the real situation suggests that the predictive efficacy of the model is excellent (**Figure 3B**). Expression distribution analysis of the identified biomarkers suggested that they are expressed at high levels in the skin of the abdomen (**Figure 3C**). In addition, *FOXF1* is expressed in endothelial cells and smooth muscle cells, *LPAR1* is expressed in endothelial cells and fibrosis, and *TRIM32* is expressed in mitotic cells (skin) (**Figure 3D**).

### 3.4 Immune cell infiltration and its relevance with biomarkers

**Figure 4:**
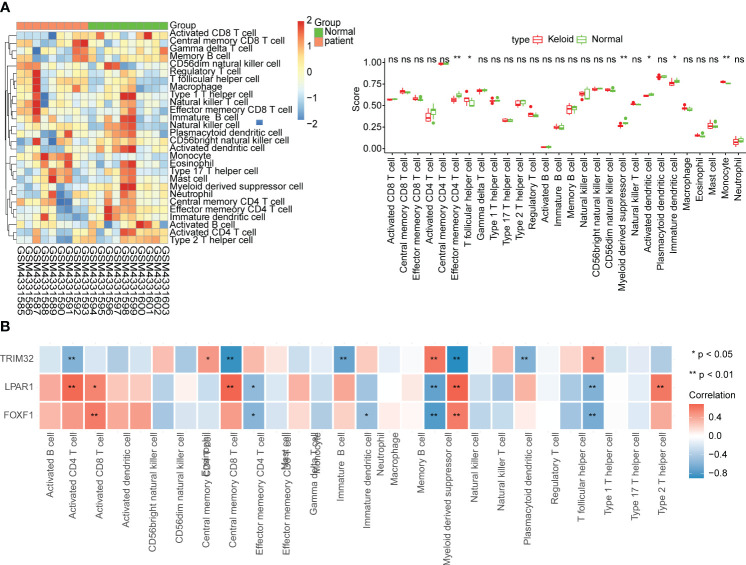
Immune infiltration analysis. **(A)** Relative abundance of immune cells and comparison between KD and normal samples. ns, not significant; *p<0.05; **p<0.01. **(B)** Correlation between biomarkers and immune cells.

Six differentially abundant immune cells were identified between the KD and control group (**Figure 4A**). Correlation analysis between the ssGSEA scores of the differentially abundant immune cells and the biomarkers suggested that *LPAR1* was positively correlated with activated CD4 T cells, myeloid-derived suppressor cells, effector memory CD4 T cells, and type 2 T helper cells (P < 0.01), *TRIM32* was positively correlated with monocytes (P < 0.01), and *FOXF1* was positively correlated with activated CD8 T cells, and myeloid-derived suppressor cells (P < 0.01) (**Figure 4B**).

### 3.5 Prediction of potential regulatory mechanisms

**Figure 5:**
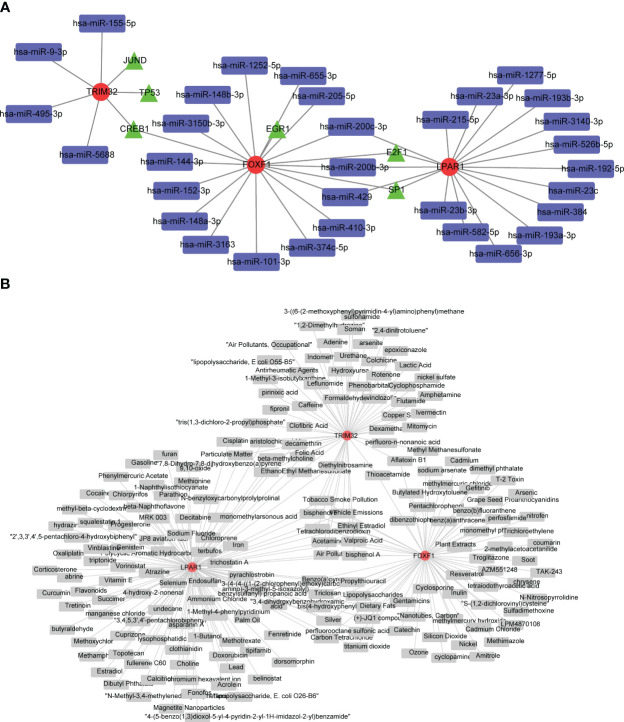
Investigation of potential regulatory mechanisms KD biomarkers, and drug predictions. **(A)** Regulatory network based on microRNAs (miRNAs), transcription factors (TFs), and biomarkers. Red circles are biomarkers, blue quadrangles are miRNAs, and green triangles are TFs. **(B)** Biomarker-drug network for KD. Red circles represent biomarkers and gray quadrangles represent drugs targeting these biomarkers.

A total of 32 miRNAs and 9 TFs were obtained and a miRNA-mRNA-TF regulatory network was constructed (**Figure 5A**; biomarkers in red, miRNAs in blue and TFs in green). *FOXF1* and *LPAR1* were regulated by E2F1 and *TRIM32* and *FOXF1* were regulated by CREB1. Sixty-seven compounds that may act on *FOXF1*, 108 compounds that may act on *LPAR1*, and 56 compounds that may act on *TRIM32* were predicted and gene-compound action networks were constructed (**Figure 5B**).

### 3.6 Expression of biomarkers in clinical samples

**Figure 6:**
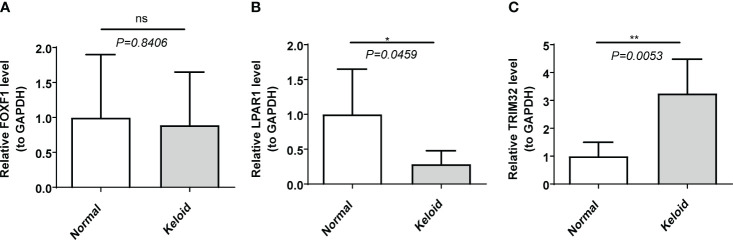
The expression of biomarkers in clinical samples by RT-qPCR. **(A)** *FOXF1*. **(B)** *LPAR1*. **(C)** *TRIM32*. ns, not significant; *p<0.05; **p<0.01.

RT-qPCR data showed that the mRNA level of *LPAR1* was significantly lower, and the mRNA level of *TRIM32* was significantly higher (P < 0.05) in the KD samples compared to the normal samples. There was no significant difference in the expression of *FOXF1* (**Figure 6**).

## 4 Discussion

KD is a benign skin tumor caused by abnormal hyperplasia of connective tissue in the skin, that occurs during prolonged abnormal wound healing. The mechanisms by which keloids form are currently unclear. Some scholars believe that the abnormal response of fibroblasts to inflammation is causes keloid formation. We propose that the inflammatory response is a significant factor in keloid pathogenesis (13–15). However, most of the current research on keloids focuses on fibroblasts and collagen with little emphasis on the importance of inflammatory genes. Therefore, finding key inflammatory genes associated with KD may help to identify new diagnostic biomarkers and drug targets.

In this study we explored the differentially expressed IRGs in two KD datasets, conducted multiple functional enrichment analyses, constructed a PPI network, and explored immune infiltration in the KD microenvironment. Finally, three keloid biomarkers were identified: *LPAR1*, *FOXF1* and *TRIM32*. In the RT-qPCR data collected from our clinical samples *LPAR1* and *TRIM32* were differentially expressed in KD samples (P<0.05) whereas *FOXF1* was not (P>0.05).

The protein encoded by *TRIM32* is a member of the tripartite motif-containing family. This protein is located in the cytoplasm and nucleus and has E3 ubiquitin ligase activity (23). TRIM32 can ubiquitinate PIAS4/PIASY and promote its degradation in UVB and TNF-α stimulated keratinocytes. In our study, the GSEA results indicated that *TRIM32* was mainly enriched in IFN-α reactions, cell apoptosis, and hypoxia. Chaudhuri et al. reported that knocking down *TRIM32* inhibited glucose-induced podocyte apoptosis, oxidative stress, and inflammatory response (24). Liu et al. reported that the gene manipulation of *Trim32* can regulate Th17 vs. Th2 immunity in response to TLR activation, suggesting that atopic dermatitis is a result of TRIM32 protein deficiency in the skin. It was speculated that *TRIM32* plays a crucial role in inflammatory diseases and congenital immunodeficiency diseases (25). Our analysis found that *TRIM32* was upregulated in publicly available KD microarray data, and RT-qPCR from our clinical samples confirmed this (P < 0.05). We speculate that *TRIM32* is closely involved in the formation of keloids. Further research on the inflammatory regulation of scarring by *TRIM32* may establish *TRIM32* as a potential treatment target for keloids.

The protein encoded by *LPAR1* is an integral membrane protein in the family of lysophosphatidic acid receptors also known as EDG receptors (26, 27). LPAR1 is involved in the reorganization, migration, differentiation, and proliferation of actin cytoskeleton, as well as its response to tissue damage and infection (28–30). *LPAR1* promotes the formation of lamellar pseudopodia at the anterior edge of migrating cells by activating RAC1. This activation plays a role in chemotaxis and cell migration, which are important in injury responses (31–33). Wu et al. reported that *LPAR1* can mediate various biological functions of tumors (34) and participate in the activation, proliferation differentiation, and migration of immune cells (32). Our correlation analysis between the ssGSEA scores of the differentially abundant immune cells and biomarkers in this study showed that *LPAR1* was positively correlated with activated CD4 T cells and effector memory CD4 T cells. *LPAR1* expression was reported to be positively correlated with the expression of chemokines and chemokine receptors, suggesting that *LPAR1* may regulate immune cell migration (35). The E2F family of transcription factors regulate cell function via gene transcription. E2F was reported as a novel fibrotic gene regulating pulmonary fibrosis (36). The enrichment of single gene GSEA in this study indicated that *LPAR1* is significantly enriched in the “E2F target” pathway. *LPAR1* is most highly expressed in endothelial cells and fibroblasts in skin and soft tissues. Our analysis showed that *LPAR1* was downregulated in KD samples, and this was confirmed by our RT-qPCR data from clinical samples. We therefore speculate that *LPAR1* plays an important inflammatory and immune regulatory role in the formation of keloids.

*FOXF1* belongs to the forkhead transcription factor family and is characterized by a unique forkhead domain (37). In an immune cell analysis of infantile angiomatosis, *FOXF1* was found to be positively correlated with the degree of monocyte infiltration (38). Recent studies have shown that overexpression of *FOXF1* can inhibit the production of α-SMA, fibronectin, and type IV collagen, thereby alleviating TGF-β1-induced fibrosis (39). In addition, overexpression of *FOXF1* can promote the proliferation of BEAS-2B cells, inhibit apoptosis, and inhibit inflammation in response to TGF-β1. Fenghua et al. reported that increasing *FOXF1* expression in endothelial cells could alleviate pulmonary fibrosis (40). *FOXF1* is highly expressed in both endothelial cells and fibroblasts, suggesting that *FOXF1* is involved in chronic inflammation following tissue injury and inhibits collagen deposition and fiber proliferation in keloid formation. In this study, GSEA results showed that *FOXF1* was mainly enriched in E2f targets, G2M checkpoints and myogenesis. However, in our RT-qPCR experiment, we found no significant difference in *FOXF1* expression between KD and normal samples (P > 0.05). This may be due to the smaller number of samples in the verification set (5 vs. 5) compared to the microarray data (10 vs. 9).

Our clinical predictive model predicts that the risk of developing KD increases as the expression of *FOXF1* and *LPAR1* decrease and the expression of *TRIM32* increases. Previous studies on these three genes support this prediction. *FOXF1* is associated with tissue development and inhibition of *FOXF1* may cause abnormalities in the cell cycle of wound tissue leading to impaired wound healing. The inhibition of *LPAR1* leads to a decrease in chemotaxis which is crucial for the inflammatory response around the wound. Moderate migration of inflammatory cells such as macrophages, mast cells, and granulocytes helps to remove necrotic cell debris and repair fibers during wound healing. Decreased expression of *LPAR1* inhibits the formation of lamellar pseudopodia at the leading edge of migrating cells which also slows down wound healing. *TRIM32* promotes the degradation of PIAS4 in keratinocytes. Therefore, increased *TRIM32* expression reduces the inhibitory effect of PIAS4 on the formation of keratinocytes, resulting in a large accumulation of keratinocytes around the wound, which secrete keratin fibers that are the main components of scar tissue.

In this study, immuno-infiltration analysis showed significant differences between keloid and normal tissue in CD4+ effector T cells, myeloid-derived suppressor cells, activated dendritic cells, immature dendritic cells, follicular helper T cells, and monocytes. The levels of CD4+ effector T cells, myeloid-derived suppressor cells, activated dendritic cells, and immature dendritic cells were significantly lower in KD tissues than in control tissues while the levels of follicular helper T cells and monocytes were significantly higher. It has been confirmed that the Th2 characteristic is possessed by KD (41). Our analysis suggested that *FOXF1* and *LPAR1* were significantly negatively correlated with monocytes and follicular helper T cells, and significantly positively correlated with myeloid-derived suppressor cells, and that the levels of monocytes and follicular T helper cells at the wound were significantly increased. Henderson et al. analyzed more than 100,000 human hepatocytes and identified a subset of macrophages associated with scarring. This group of macrophages express *TRIM32* and *CD9*, are differentiated from circulating monocytes, and are known to promote fibrosis (42). Previous studies have reported that monocytes and macrophages are key components of the immune system and participate in the regulation of inflammatory immunity and tissue repair by activating T and B lymphocytes (43). Follicular helper T cells are involved in the humoral immune regulation of inflammation and play a crucial role in autoimmunity and tumor-related immunity (44). Myeloid-derived suppressor cells are a group of suppressor cells of bone marrow origin which are precursors of dendritic cells, macrophages, and granulocytes, and have the ability to significantly inhibit immune cell responses (45). Chronic inflammation and fibrosis may be caused by improper activation of the immune response mediated by macrophages, an example of which is the development of fibrosis in systemic sclerosis (46). The biomarkers we identified are related to monocytes, myeloid-derived suppressor cells, and follicular helper T cells, which may all play an important role in the formation of keloids.

In the immune infiltration analysis, we found that there were different degrees of correlation between the biomarkers and the infiltration of immune cells. In order to further explore the role of immune cells in the development of KD, we used the HPA database to explore the expression of the biomarkers in different cell types. LPAR1 was enriched in macrophages, T-cells and mast cells, TRIM32 was enriched in T cells and mast cells, while FOXF1 was not significantly expressed in any immune cells. In a mouse model of multiple sclerosis, Choi et al. found that LPAR1-3 antagonists increased cell infiltration and immune cell activation (including macrophages) (PMID:34666785). In addition, Choi et al. demonstrated that in the immune microenvironment of tumors, different LPA receptors promoted metastasis, which helped create a T cell rejection and pro-tumor microenvironment suitable for therapeutic intervention (PMID: 34788605). Wang et al. reported that in a mouse model of atopic dermatitis (AD), TRIM32 acted as a regulator of PKCζ and could control the differentiation of Th2 cells, which are very important for the pathogenesis of AD (PMID: 33096083). We believe that immune cells, in particular T cells, play an important role in the development of KD, and are expected to become a new target for KD immunotherapy. However, the molecular mechanisms involved need further investigation.

In this study the transcriptional regulatory network analysis indicated that *FOXF1* and *LPAR1* share two transcriptional regulatory factors, E2F1 and SP1. In addition, the two share three miRNAs, hsa-miR-200c-3p, hsa-miR-200b-3p, and hsa-miR-429. The downregulation of *FOXF1* and *LPAR1* in keloid patients could be caused by the inactivation of E2F1 and SP1 due to mutations or other factors, or by the effect of the three miRNAs. *TRIM32* did not share any miRNAs with the other two genes. JUND and TP53 were predicted to target *TRIM32*, which may contribute to its upregulation.

This study has several limitations. First, our analysis was based on a limited number of clinical samples from public databases, and may suffer from poor statistical power due to the small sample size. In addition, our analysis of the expression patterns of the identified biomarkers was based on public databases, and further validation is necessary, which would need to be done by collecting a larger number of clinical samples or conducting animal experiments. Given these limitations, larger datasets are needed to support further research and validation of the genes and molecular mechanisms that we identified.

In this article we analyzed IRGs in KD, leading to the identification of two new biomarkers of keloid tissue. Further studies on IRGs in KD may lead to new tools for early diagnosis as well as the identification of novel drug targets for treatment of KD.

## Data availability statement

The datasets presented in this study can be found in online repositories. The names of the repository/repositories and accession number(s) can be found in the article/**Supplementary Material**.

## Ethics statement

The studies involving humans were approved by Research Ethics committee of the second hospital of shandong university. The studies were conducted in accordance with the local legislation and institutional requirements. The participants provided their written informed consent to participate in this study.

## Author contributions

XCW: Data curation, Methodology, Validation, Writing – original draft, Writing – review & editing. XYW: Data curation, Validation, Writing – review & editing. ZL: Methodology, Resources, Writing – review & editing. LL: Data curation, Resources, Writing – review & editing. JZ: Methodology, Resources, Writing – review & editing. DJ: Data curation, Writing – review & editing, Methodology. GH: Methodology, Writing – review & editing, Resources.
