# Supplementary material for: Identification of inflammation-related biomarkers in keloids
Source: Front Immunol. 2024 Feb 20;15:1351513. doi: 10.3389/fimmu.2024.1351513 (PMC10912164; doi:10.3389/fimmu.2024.1351513)
Supplement: Supplementary file 2 [file DataSheet_1.docx]

Supplementary Material

# Supplementary Figure


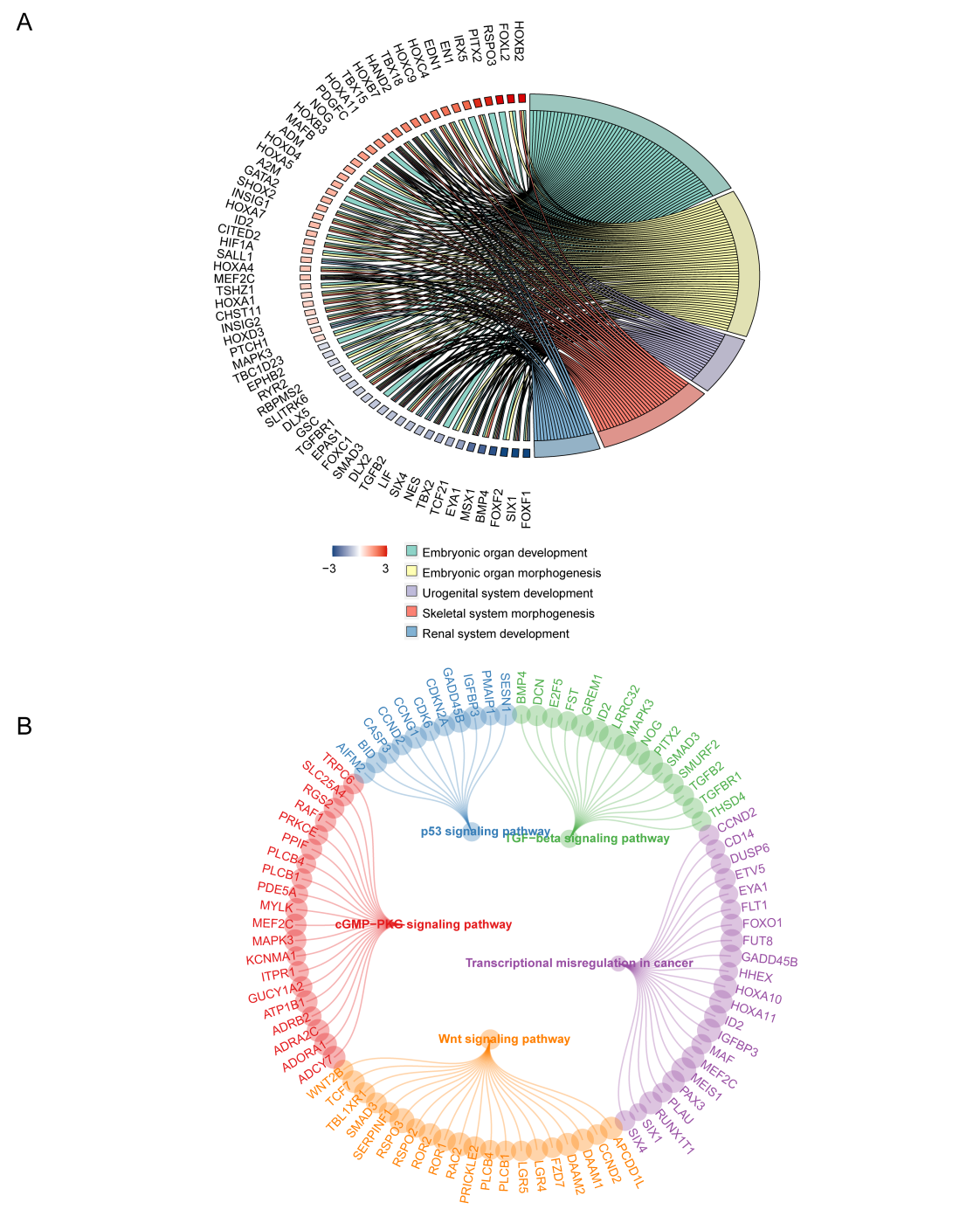


**Supplementary Figure 1.** Functional enrichment analysis of differentially expressed genes (DEGs) in the GSE145725 dataset. **(A,B)** The top5 Gene Ontology (GO) terms **(A)** and Kyoto Encyclopedia of Genes and Genomes (KEGG) pathways **(B)** enriched in DEGs.
