# Supplementary figures and images for: Identification of inflammation-related biomarkers in keloids
Source: Front Immunol. 2024 Feb 20;15:1351513. doi: 10.3389/fimmu.2024.1351513 (PMC10912164; doi:10.3389/fimmu.2024.1351513)

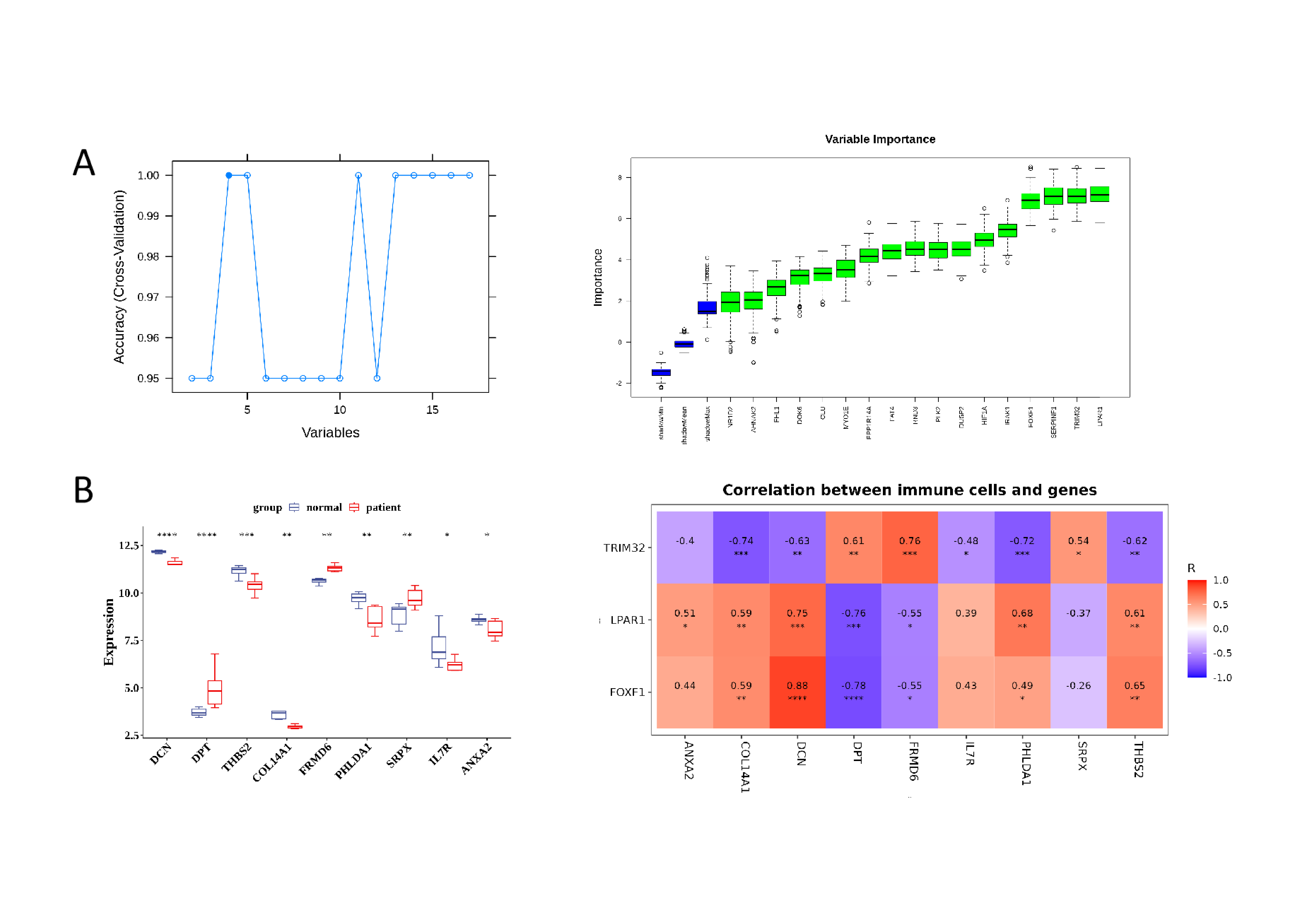

Supplement: Supplementary file 3 [file Image_2.tif]
